# Supplementary material for: MetaRibo-Seq measures translation in microbiomes
Source: Nat Commun. 2020 Jun 29;11:3268. doi: 10.1038/s41467-020-17081-z (PMC7324362; doi:10.1038/s41467-020-17081-z)
Supplement: Supplementary file 10 — Supplementary Data 7 [file 41467_2020_17081_MOESM10_ESM.zip › File2/Confidence_High_Taxonomy/202897_out.krona.html]

Javascript must be enabled to view this page.

members
magnitude
magnitudeUnassigned
count
unassigned
taxon
rank

202897\_out

12

2
12
superkingdom

phylum
12
1239

class
12
186801

12
186802
order

family
541000
12

12
1263
genus

165186

SRS014923\_contig\_number\_46451SRS050925\_contig\_number\_30317
2
species

2293157
1

SRS077502\_contig\_number\_contig-100\_33.112897
species

41978
6

SRS014979\_contig\_number\_26007SRS049446\_contig\_number\_13272SRS065504\_contig\_number\_34044SRS098073\_contig\_number\_4457SRS144183\_contig\_number\_30994SRS149325\_contig\_number\_14733
species

2293156
1

SRS150029\_contig\_number\_1702
species

species

SRS1055056\_contig\_number\_1820SRS893341\_contig\_number\_contig-100\_789.83590
2
1262962
